# Supplementary figures and images for: Cryo-electron tomography of Birbeck granules reveals the molecular mechanism of langerin lattice formation
Source: eLife. 2022 Jun 27;11:e79990. doi: 10.7554/eLife.79990 (PMC9259017; doi:10.7554/eLife.79990)

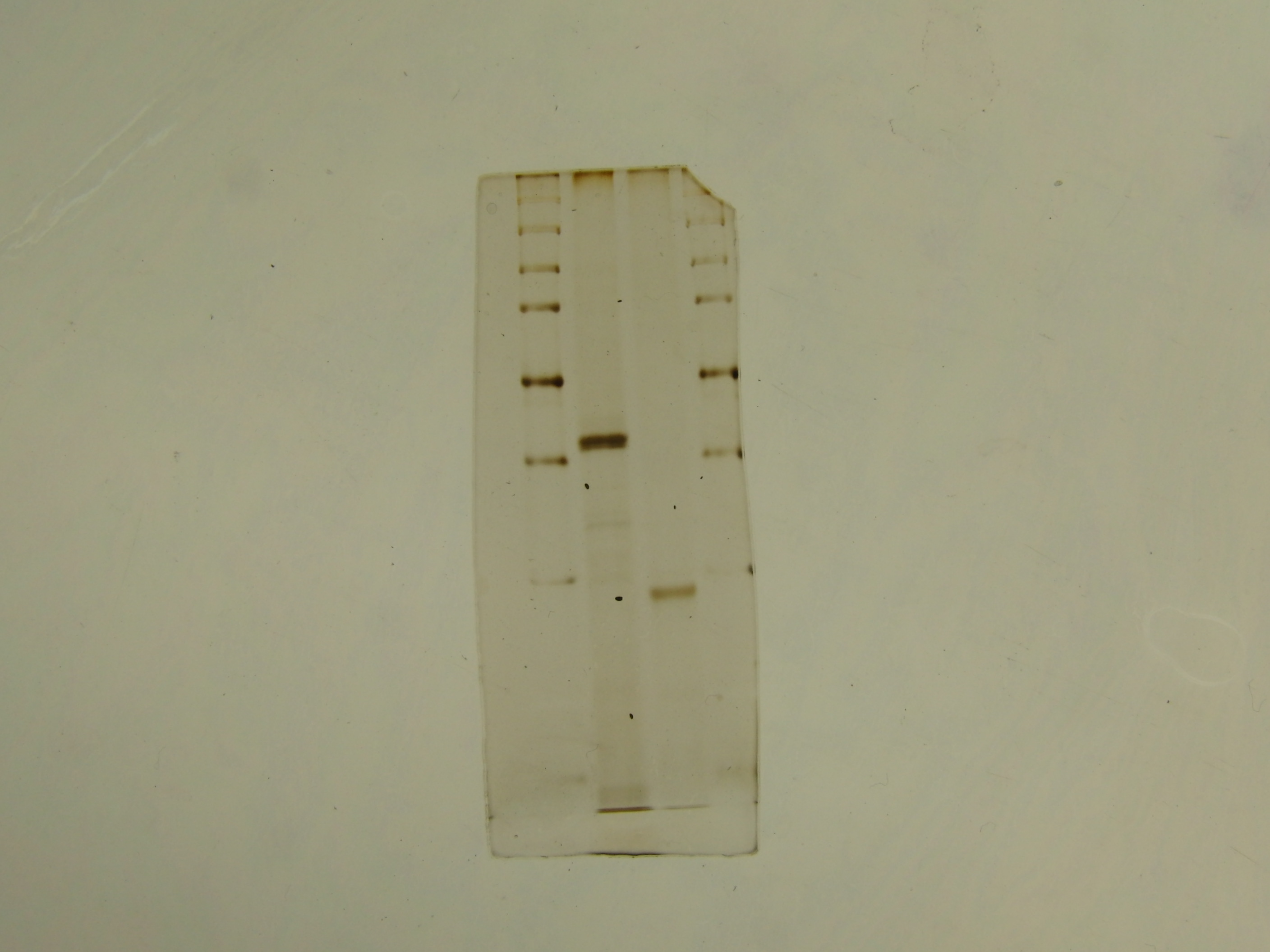

Supplement: Figure 1—source data 1. [file elife-79990-fig1-data1.zip › Figure1-source data1.JPG]

**Original gel image: Figure 1D**

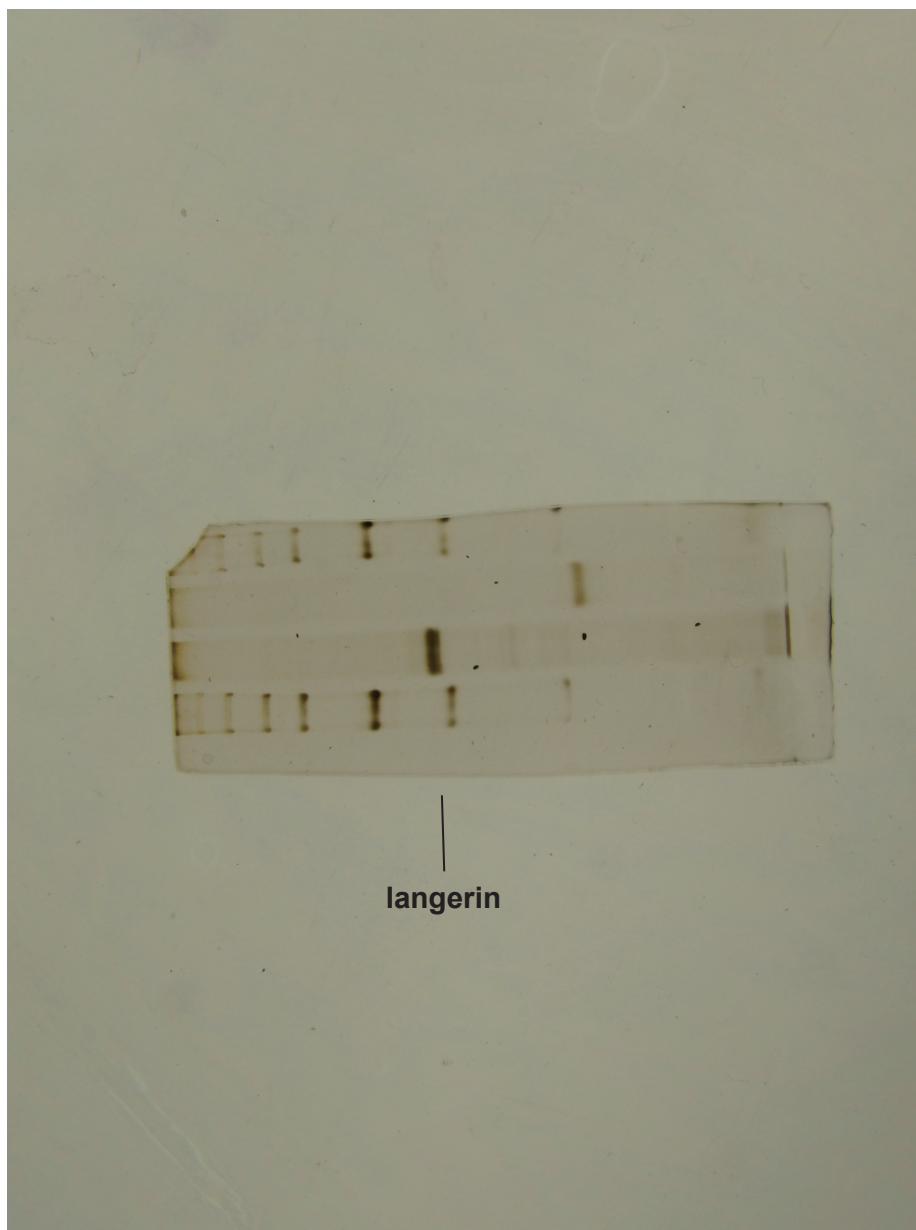

Supplement: Figure 1—source data 2. [file elife-79990-fig1-data2.pdf]

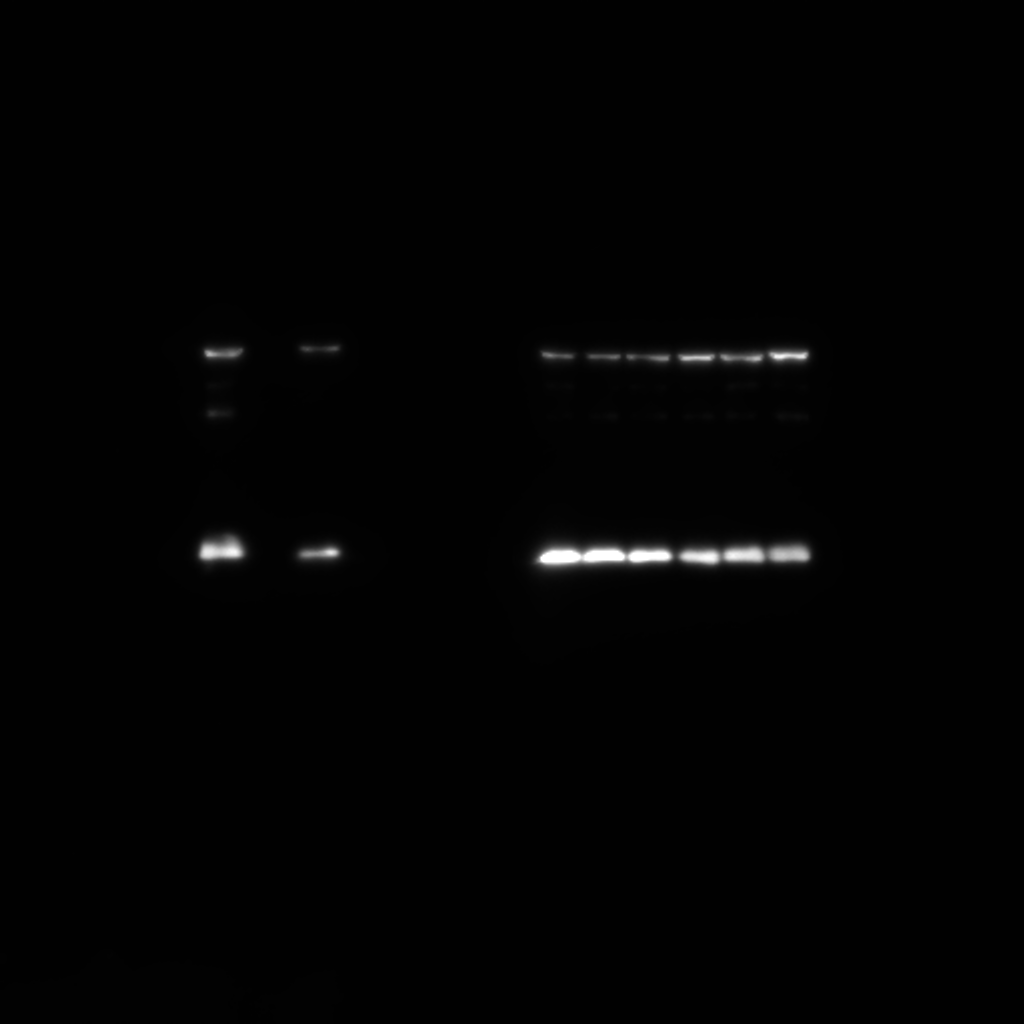

Supplement: Figure 5—source data 1. [file elife-79990-fig5-data1.zip › Figure5-source data 1.Tif]

Original western blot file: Figure 5B(right)

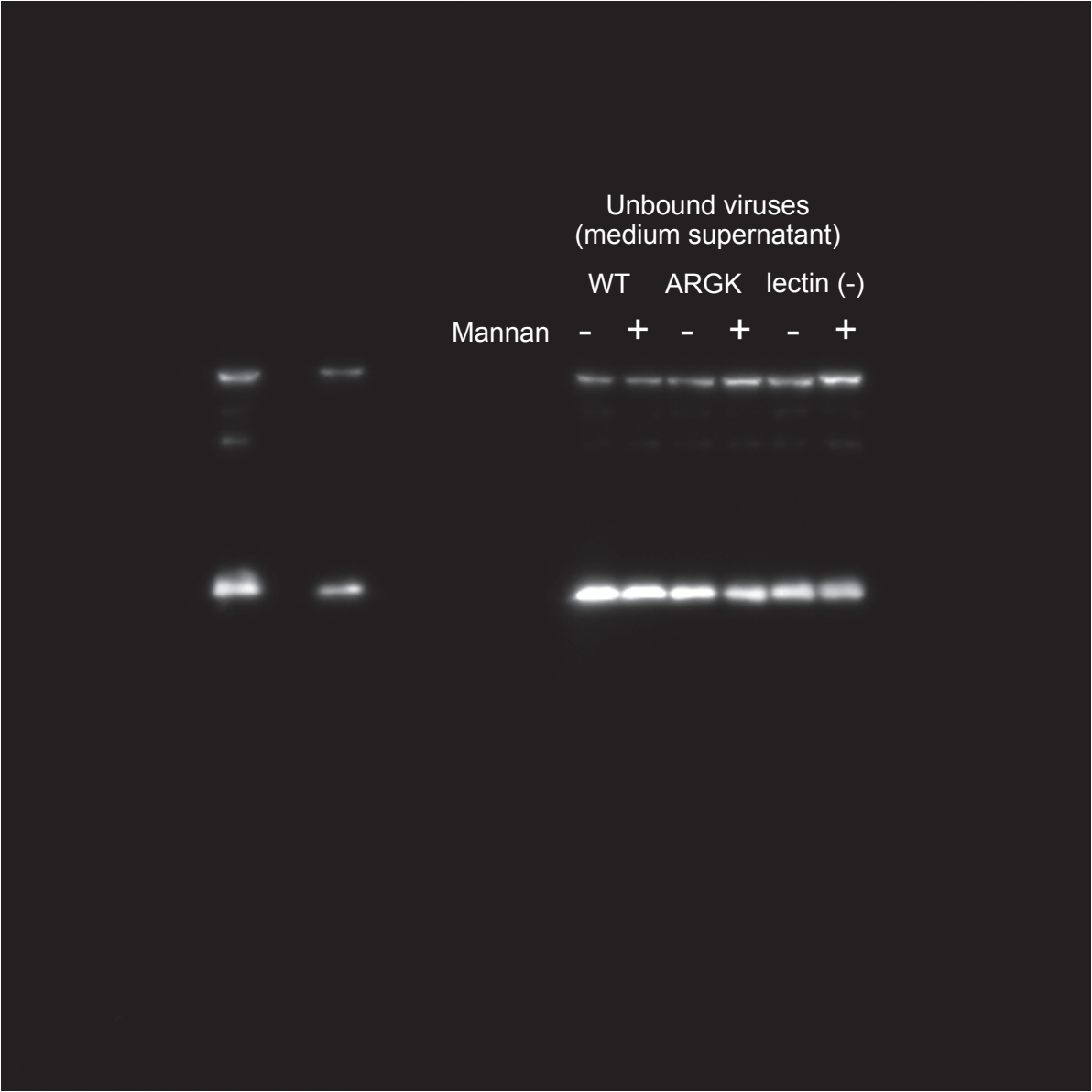

Supplement: Figure 5—source data 2. [file elife-79990-fig5-data2.pdf]

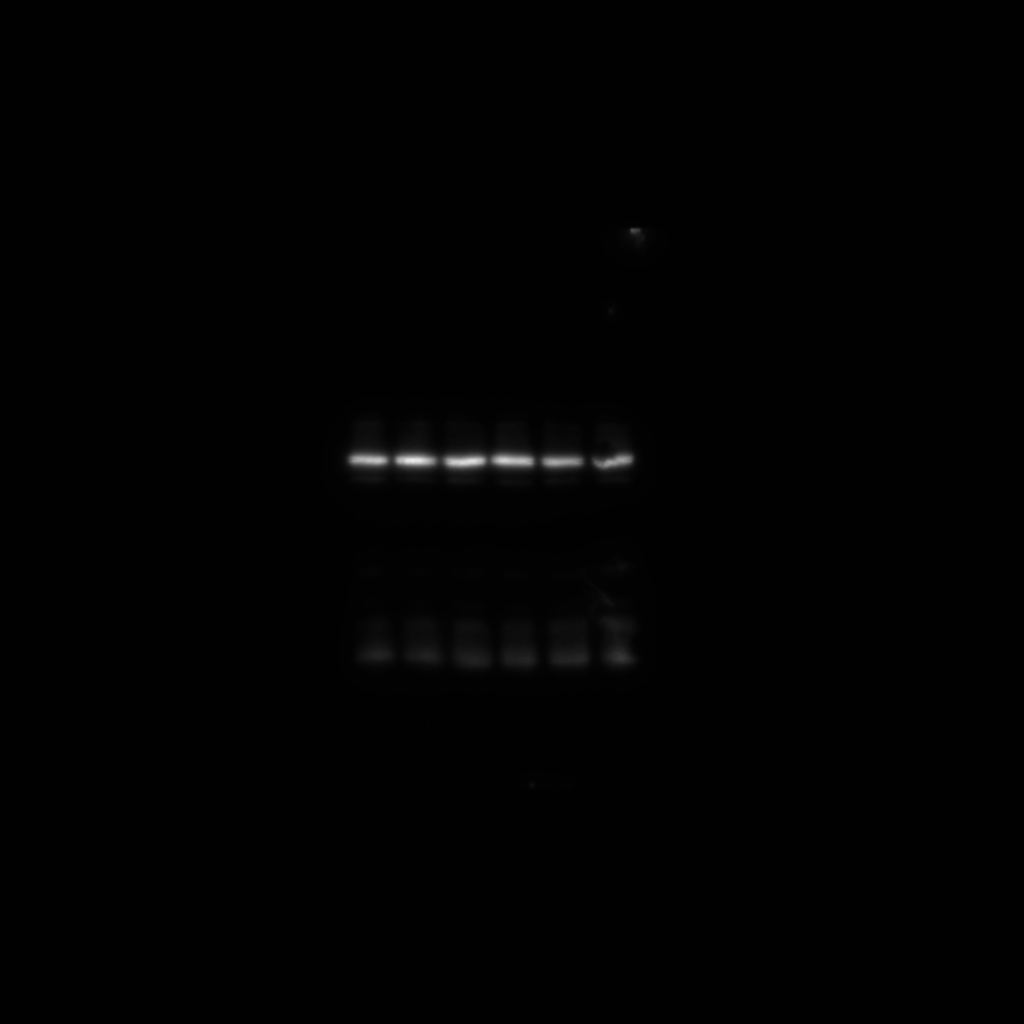

Supplement: Figure 5—source data 3. [file elife-79990-fig5-data3.zip › Figure5-source data 3.Tif]

Original western blot file: Figure 5B

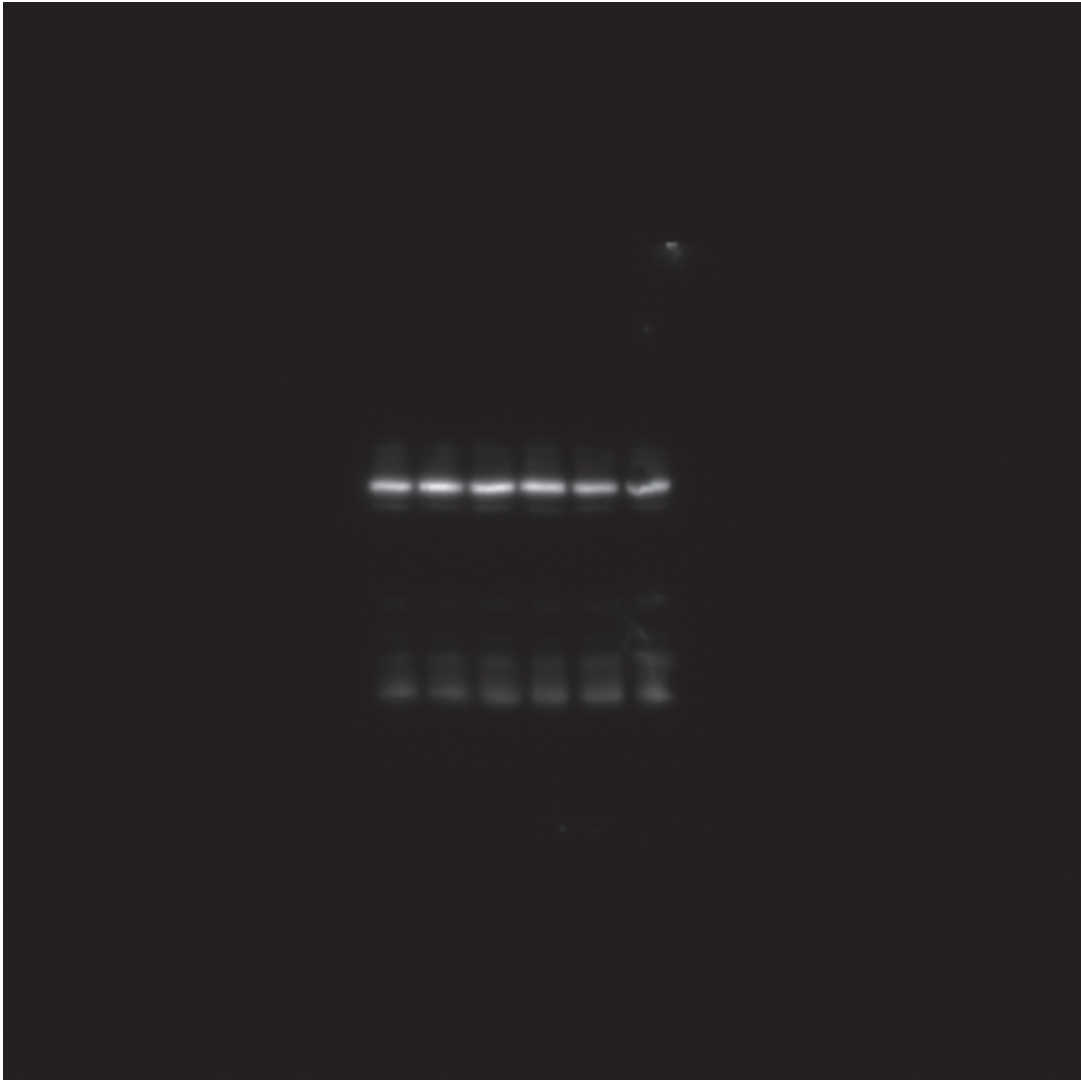

langerin

Supplement: Figure 5—source data 4. [file elife-79990-fig5-data4.pdf]

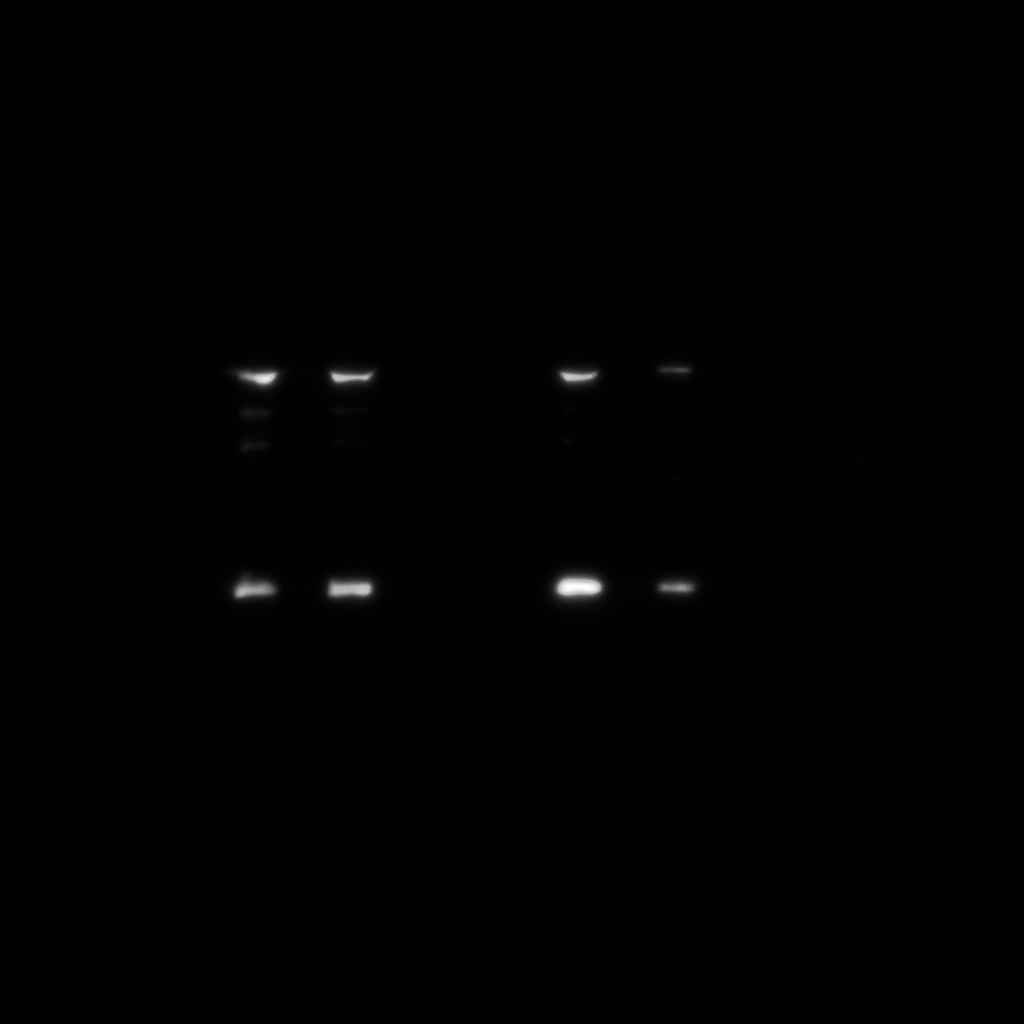

Supplement: Figure 5—source data 5. [file elife-79990-fig5-data5.zip › Figure5-source data 5.Tif]

Original western blot file: Figure 5B(left) and 5C

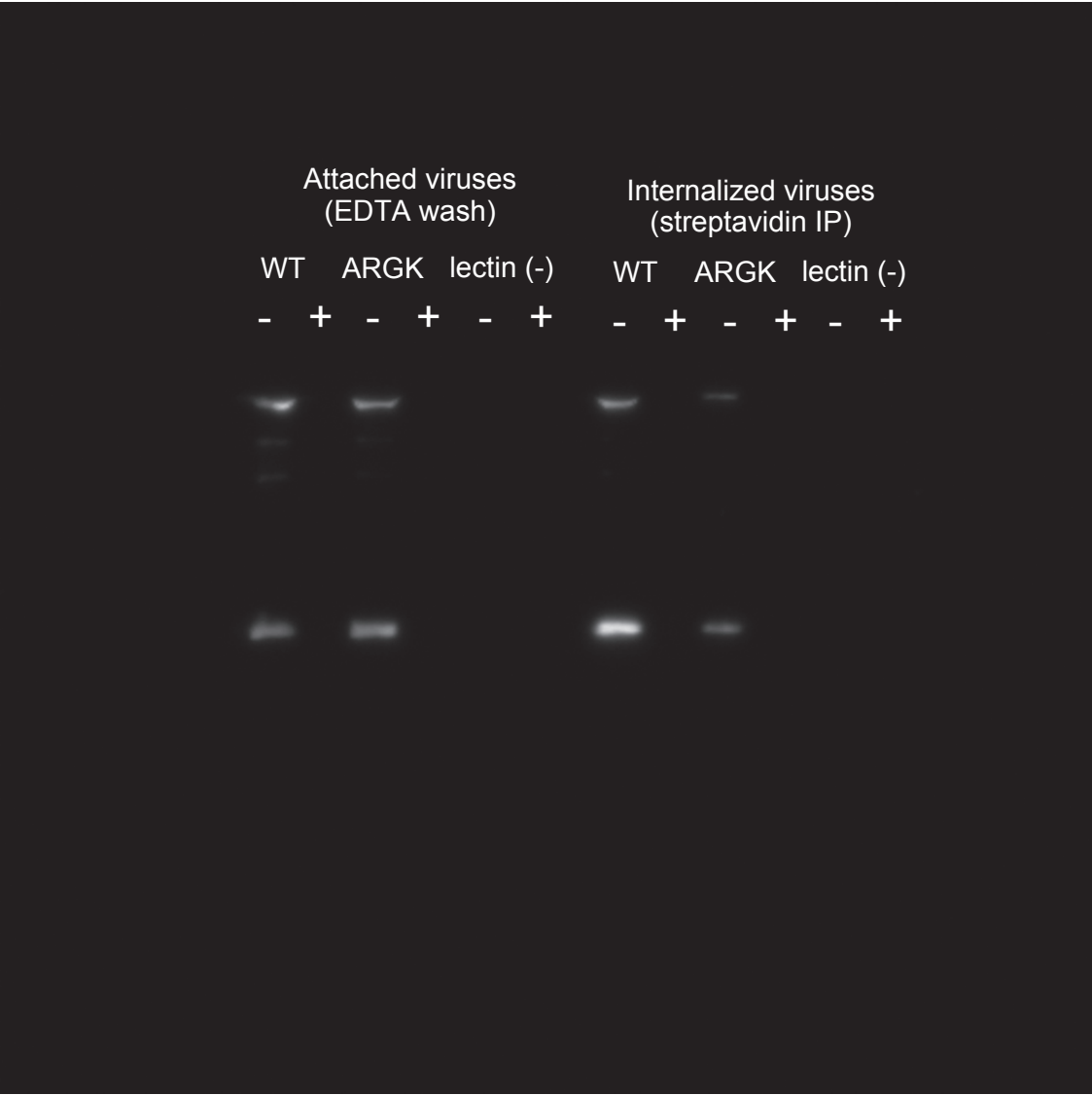

Supplement: Figure 5—source data 6. [file elife-79990-fig5-data6.pdf]

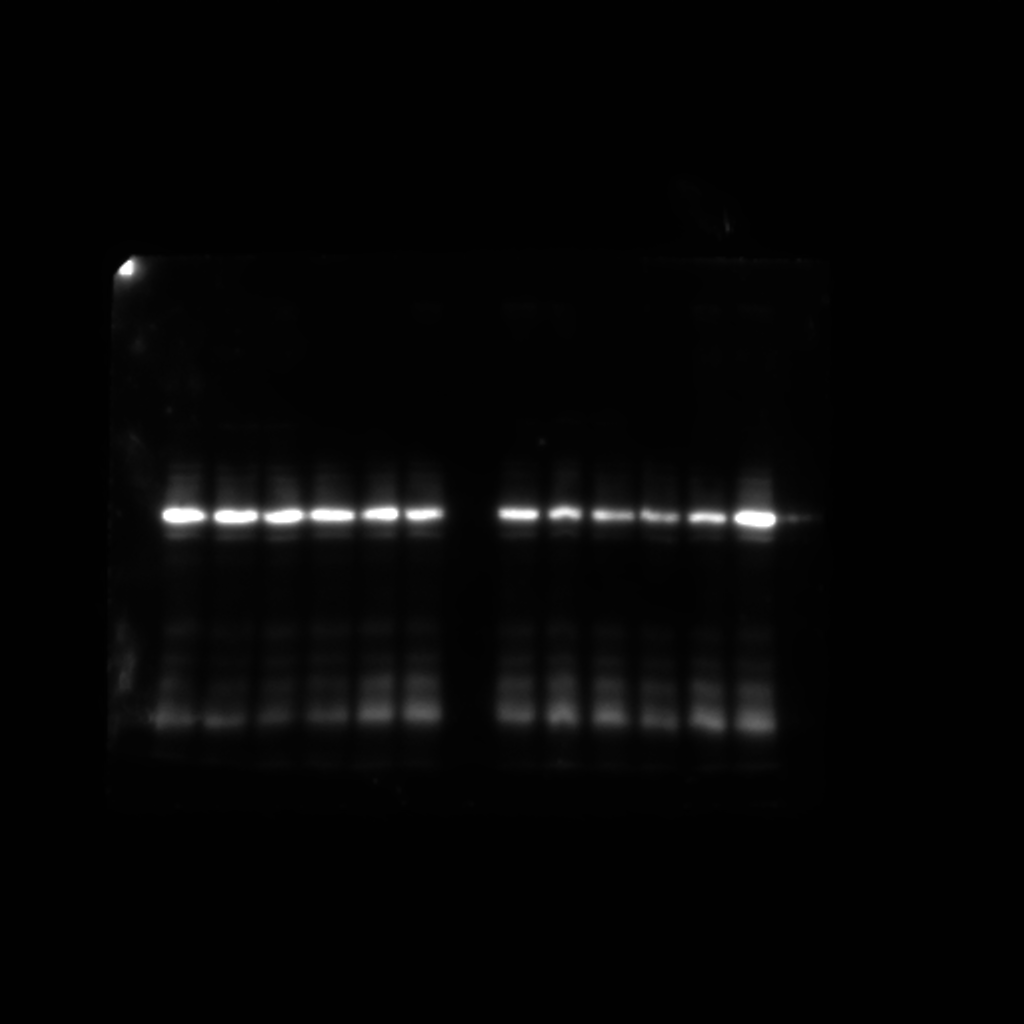

Supplement: Figure 5—source data 7. [file elife-79990-fig5-data7.tif]

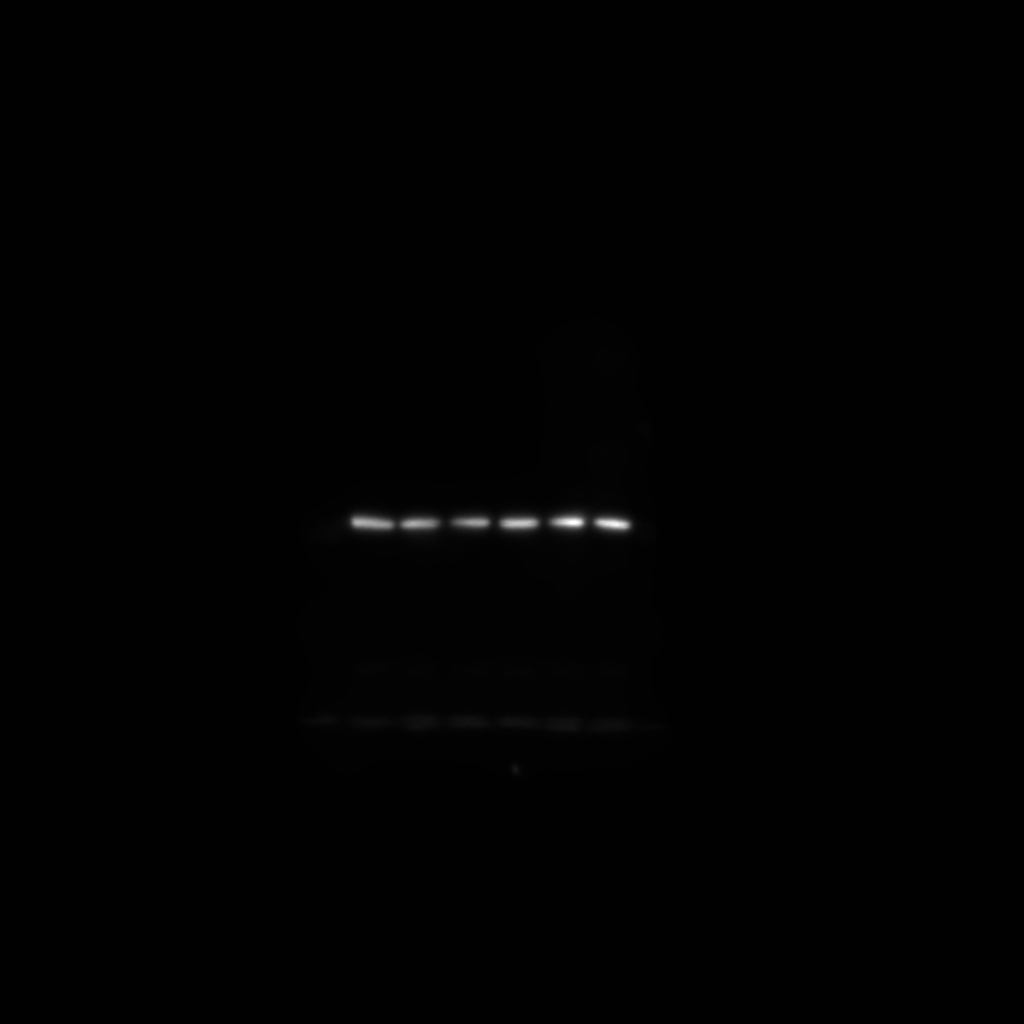

Supplement: Figure 5—source data 8. [file elife-79990-fig5-data8.zip › Figure5-source data 8.Tif]

Original western blot file: Figure 5C (bottom)

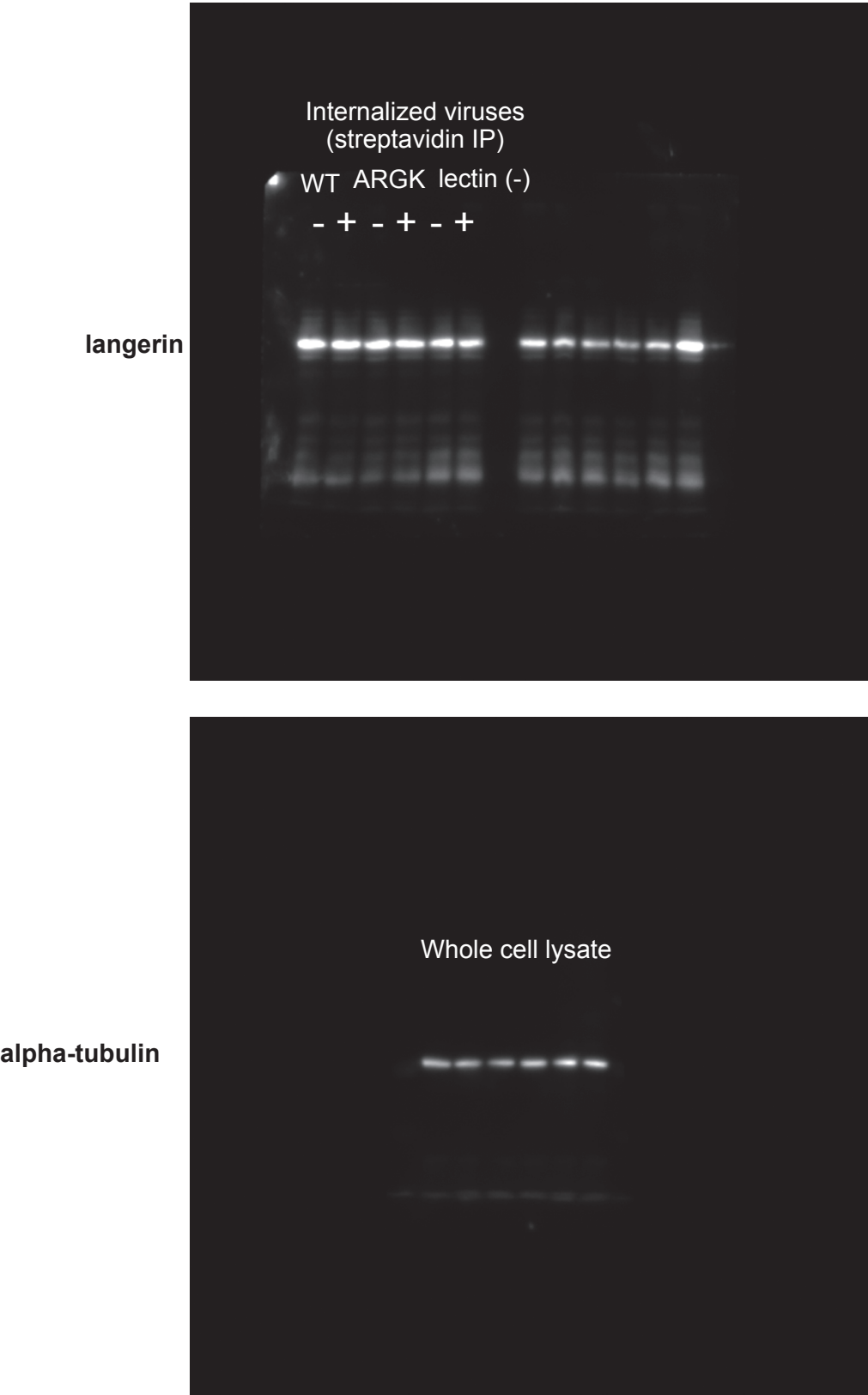

Supplement: Figure 5—source data 9. [file elife-79990-fig5-data9.pdf]

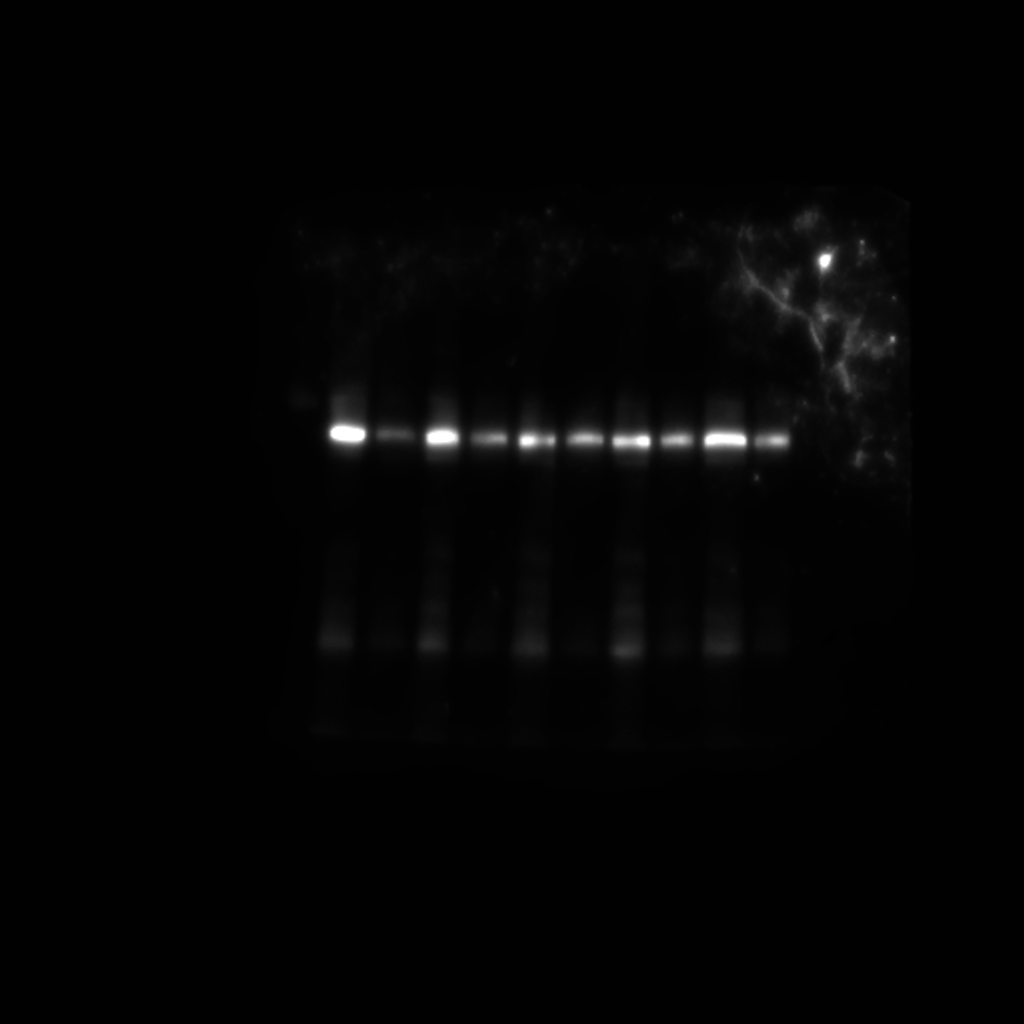

Supplement: Figure 5—figure supplement 1—source data 1. [file elife-79990-fig5-figsupp1-data1.zip › Figure5-figure supplement 1-source file 1.Tif]

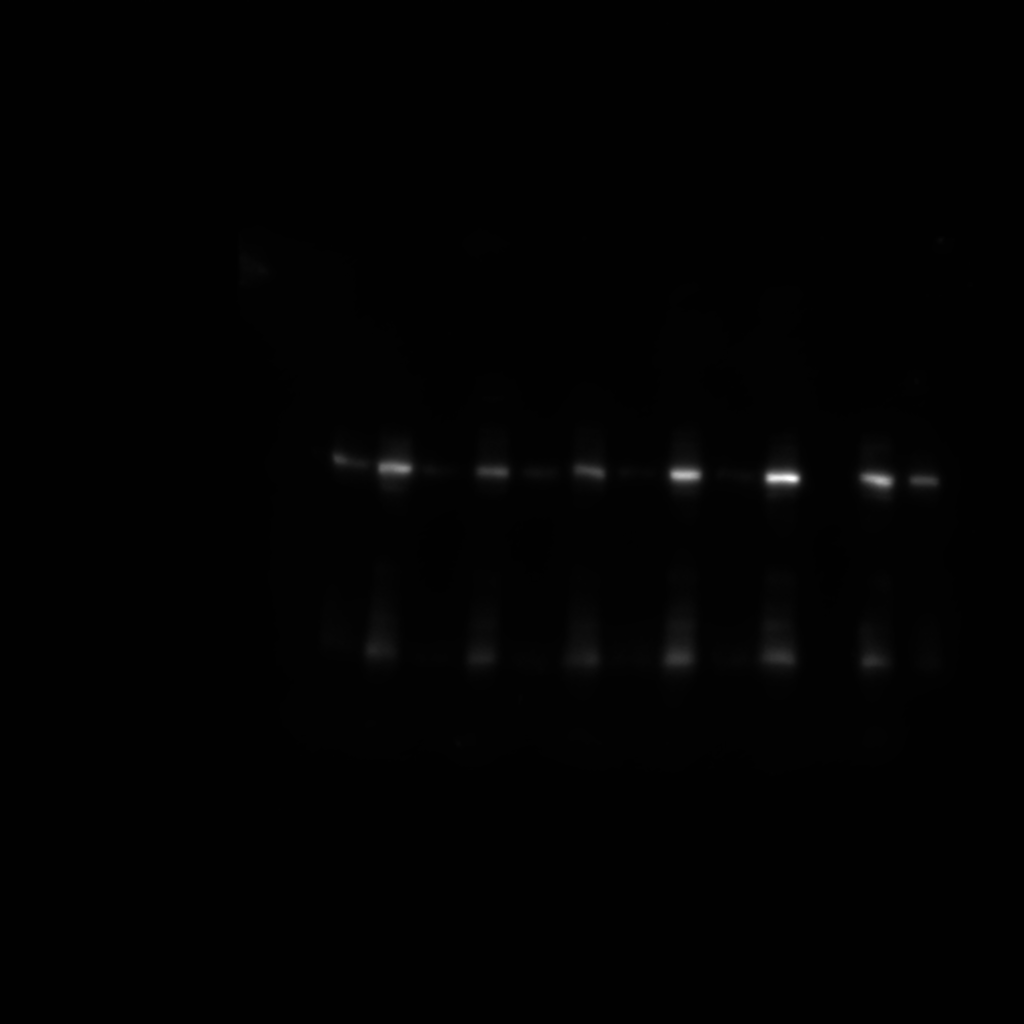

Supplement: Figure 5—figure supplement 1—source data 3. [file elife-79990-fig5-figsupp1-data3.zip › Figure5-figure supplement 1-source file 3.Tif]

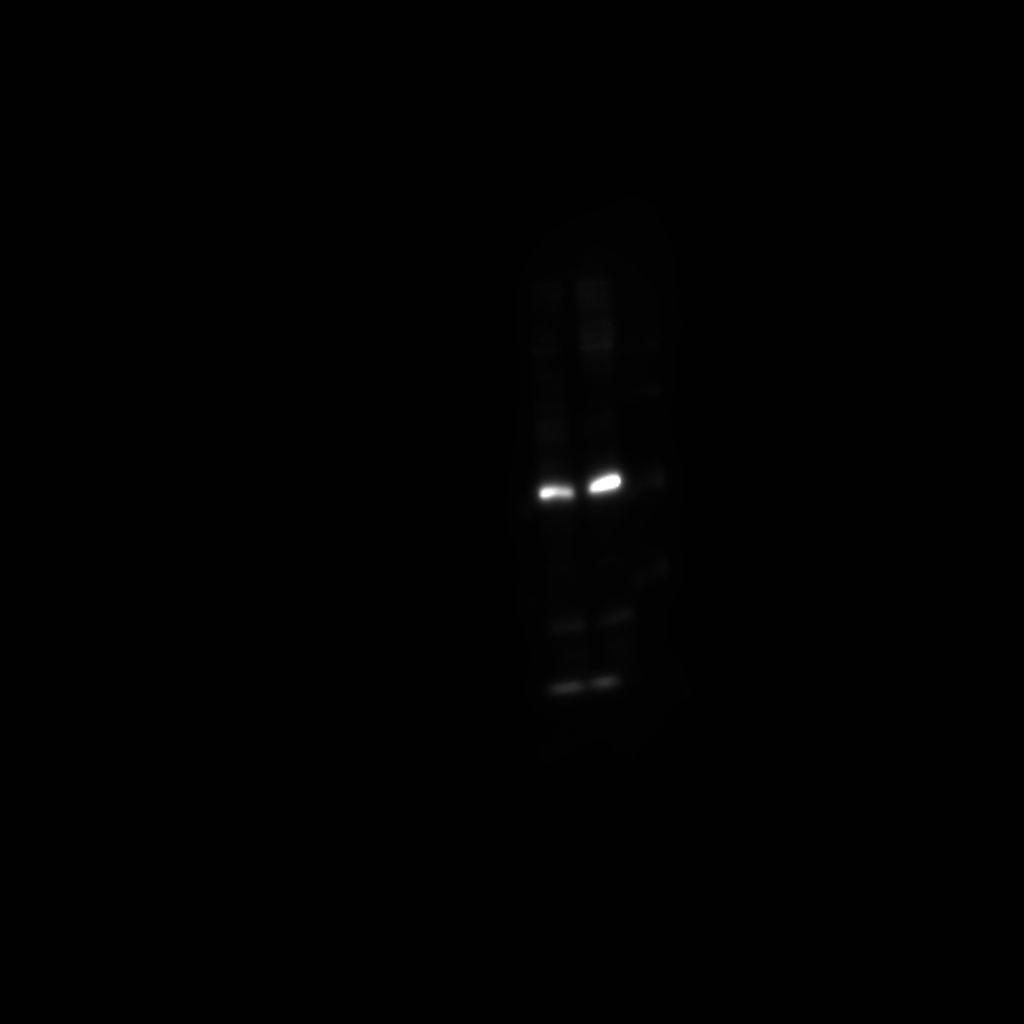

Supplement: Figure 5—figure supplement 1—source data 4. [file elife-79990-fig5-figsupp1-data4.zip › Figure5-figure supplemen 1-antitubulin.Tif]

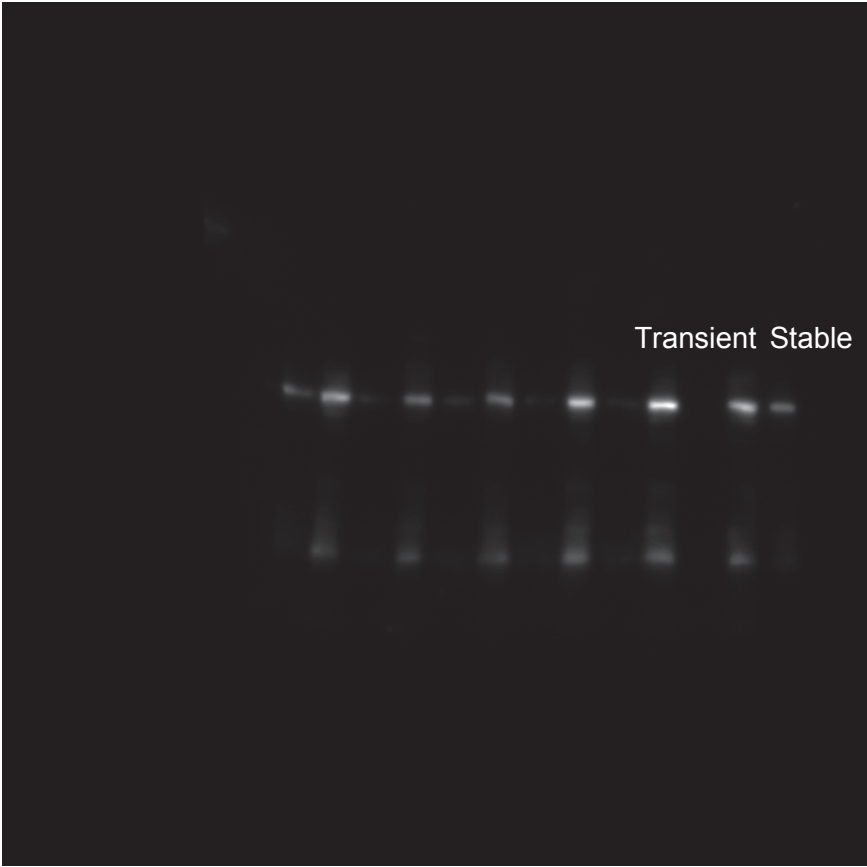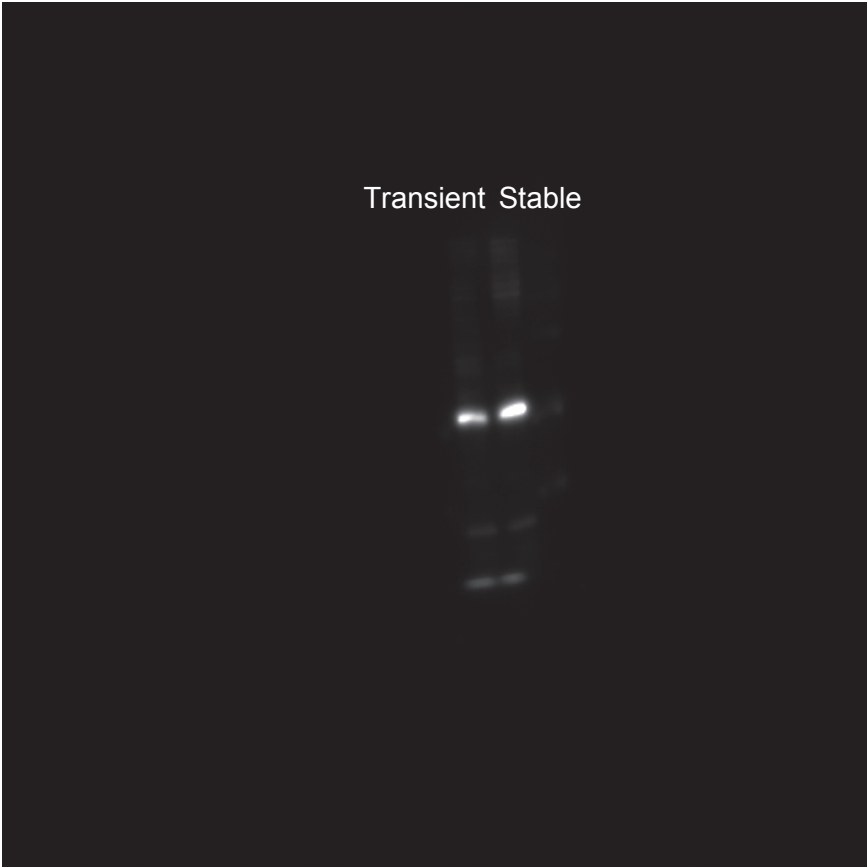

Supplement: Figure 5—figure supplement 1—source data 4. [file elife-79990-fig5-figsupp1-data4.zip › Figure5-figuresupplement1C-antilangerin_and_tubulin.pdf]
