# Supplementary material for: Cryo-electron tomography of Birbeck granules reveals the molecular mechanism of langerin lattice formation
Source: eLife. 2022 Jun 27;11:e79990. doi: 10.7554/eLife.79990 (PMC9259017; doi:10.7554/eLife.79990)

Original western blot file: Figure 5-figure supplement 1A

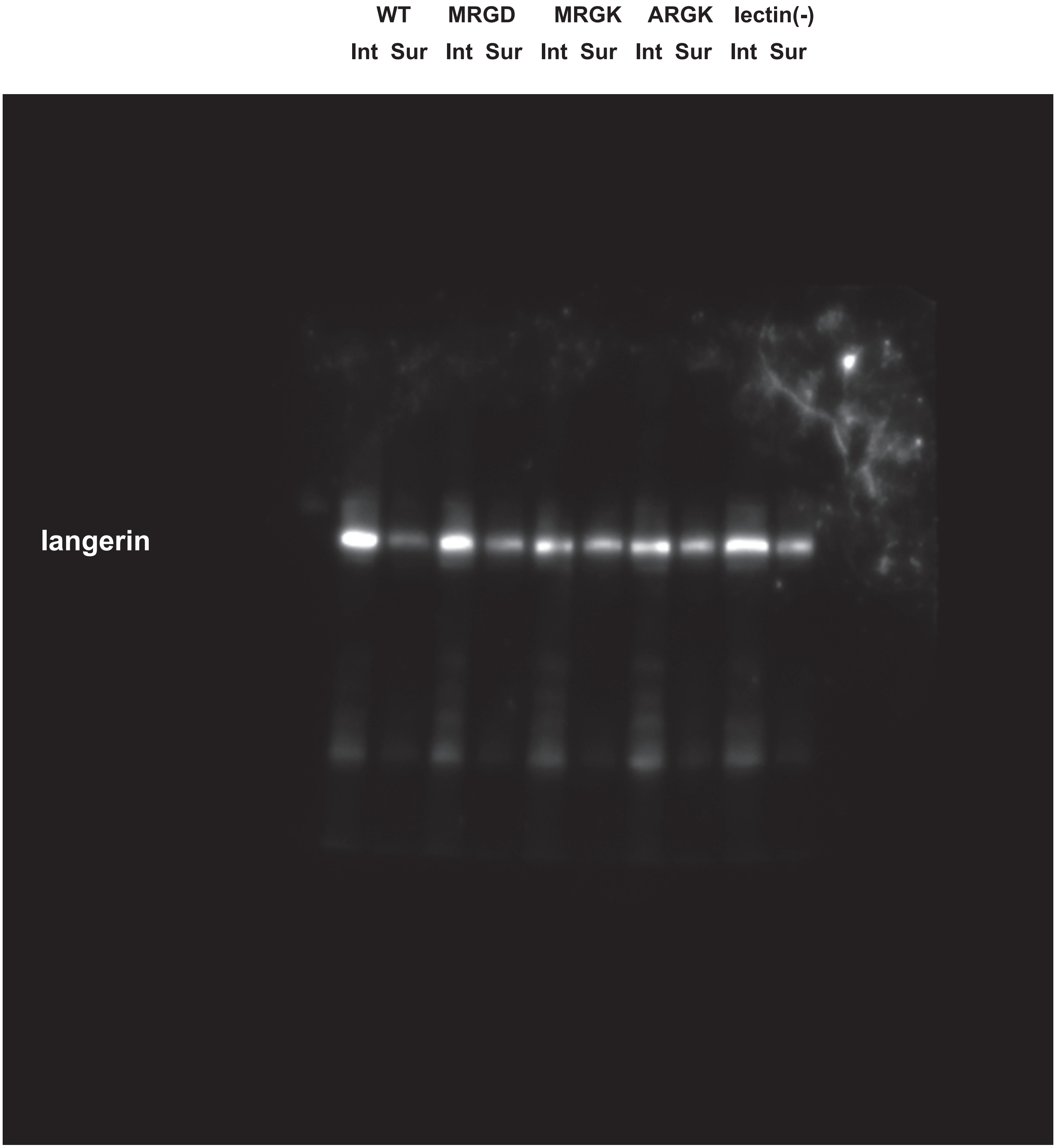

Supplement: Figure 5—figure supplement 1—source data 2. [file elife-79990-fig5-figsupp1-data2.pdf]
