# Supplementary material for: Cryo-electron tomography of Birbeck granules reveals the molecular mechanism of langerin lattice formation
Source: eLife. 2022 Jun 27;11:e79990. doi: 10.7554/eLife.79990 (PMC9259017; doi:10.7554/eLife.79990)
Supplement: Supplementary file 2. [file elife-79990-supp2.docx]

**Supplementary File 1: Summary of data collection and model validation**

| **Data collection parameters** |  |
| --- | --- |
| Magnification | 33,000× |
| Pixel size (Å) | 2.67 |
| Defocus range (µm) | 2.6 – 8.5 |
| Voltage (keV) | 300 |
| Exposure time (sec/frame) | 0.74 |
| Number of frames per tilt | 20 |
| Angular range (°) | ±60 |
| Increments (°) | 3 |
| Total dose (e^-^/Å^2^) | 49.6 |
| Box size (pixels) | 128 |
| Number of tilt series recorded | 126 |
| Number of tilt series processed | 33 |
| Initial number of subtomograms | 93,953 |
| Final number of subtomograms | 63,563 |
| **Model validation statistics** |  |
| Initial model used (PDB ID) | 3KQG |
| Bonds length RSMD (Å) | 0.003 |
| Bonds angles RSMD (°) | 0.651 |
| MolProbability score | 1.83 |
| Clash score | 21.13 |
| Rotamer outliers (%) | 0.0 |
| Ramachandran plot (%) | Favored: 97.94  Allowed: 2.06  Outliers: 0.00 |
| CaBLAM outliers (%) | 1.14 |
| B-factors (min/max/mean) | 443.64/1003.68/628.65 |
| Map resolution estimates (Å) | 6.4 (FSC_half-map_=0.143)  8.5 (FSC_model_=0.143) |
